# Initial Negative MRI Spine and Persistent Staphylococcus aureus Bacteraemia Lead to Delayed Diagnosis of Lumbar Spondylodiscitis and Epidural Abscess: A Case Report

**DOI:** 10.7759/cureus.114353

**Published:** 2026-08-11

**Authors:** Mina Zaki, Taha Yaseen, Alisha Noureen Rafeek

**Affiliations:** 1 Acute Medicine, County Durham and Darlington NHS Foundation Trust, Darlington, GBR; 2 Internal Medicine, County Durham and Darlington NHS Foundation Trust, Darlington, GBR

**Keywords:** infective discitis, mid back pain, spinal cord infection, staph aureus bacteremia, vertebral discitis

## Abstract

Vertebral osteomyelitis and spondylodiscitis are uncommon but potentially life-threatening infections that may present with non-specific symptoms, resulting in delayed diagnosis. We report the case of a 65-year-old man who presented with acute low back pain, high-grade fever, confusion, and transient neurological symptoms. Initial investigations demonstrated markedly elevated inflammatory markers and persistent methicillin-sensitive *Staphylococcus aureus* (MSSA) bacteremia.

A CT of the head and thorax/abdomen/pelvis, transthoracic echocardiography, and an early whole-spine MRI study failed to identify the source of infection. Despite appropriate intravenous antimicrobial therapy, the patient remained bacteraemic with persistent back pain, prompting repeat MRI, which demonstrated L5-S1 spondylodiscitis complicated by an anterior epidural abscess causing cauda equina compression and an associated prevertebral collection. As the patient remained neurologically intact, a multidisciplinary team involving microbiology, radiology, cardiology, and spinal surgery agreed on conservative management with prolonged intravenous flucloxacillin. The patient demonstrated progressive clinical improvement with resolution of fever, declining inflammatory markers, preserved neurological function, and successful discharge to complete outpatient parenteral antimicrobial therapy.

This case highlights the importance of maintaining a high index of suspicion for vertebral infection in patients with persistent *S. aureus* bacteremia (SAB) and ongoing back pain despite initially negative imaging. It also emphasises the value of repeat MRI when clinical suspicion persists and demonstrates that carefully selected patients with spinal epidural abscess may be successfully managed without surgery through a multidisciplinary approach.

## Introduction

Vertebral osteomyelitis and spondylodiscitis are uncommon but serious infections, accounting for approximately 3% to 5% of all cases of osteomyelitis [1]. Their incidence has increased over recent decades owing to an aging population, greater prevalence of comorbidities, and improved diagnostic imaging. *Staphylococcus aureus *remains the most common causative organism [2], with hematogenous spread representing the principal route of infection. Delayed diagnosis is common because patients often present with non-specific symptoms such as back pain and fever, while neurological deficits may be absent during the early stages of disease [1,2].

Persistent *S. aureus* bacteremia (SAB) [1-3] should prompt a thorough search for metastatic foci of infection, particularly involving the spine and heart. The MRI is the imaging modality of choice for diagnosing vertebral osteomyelitis and spinal epidural abscess [4]. However, MRI performed early in the disease course may be falsely negative or demonstrate only subtle inflammatory changes before the characteristic imaging features become apparent. Consequently, repeat contrast-enhanced MRI should be considered within one to three weeks when clinical suspicion remains high despite an initially non-diagnostic examination [5].

The management of spinal epidural abscesses remains challenging and often requires a multidisciplinary approach involving infectious diseases or microbiology, radiology, spinal surgery, and cardiology. Although urgent surgical decompression is recommended for patients with neurological deficits or spinal instability, carefully selected patients without neurological compromise may be successfully managed with prolonged targeted antimicrobial therapy alone.

We present a case of persistent methicillin-sensitive *S. aureus* (MSSA) bacteremia in a patient with severe low back pain whose initial imaging failed to identify a spinal source of infection. Repeat MRI subsequently demonstrated L5-S1 spondylodiscitis complicated by an anterior epidural abscess compressing the cauda equina. This case highlights the importance of maintaining a high index of suspicion, repeating spinal imaging when clinical suspicion persists, and the role of multidisciplinary decision-making in the successful conservative management of spinal epidural abscess.

## Case presentation

A 65-year-old man with a past medical history of essential hypertension, non-diabetic hyperglycemia, gastro-oesophageal reflux disease, and a sliding hiatus hernia presented to the emergency department with a five-day history of progressively worsening lower back pain and a two-day history of fever. The back pain had started suddenly on waking, without preceding trauma, and was described as constant, dull, and exacerbated by movement, particularly when attempting to get out of bed or roll over. The pain was partially relieved by analgesia. He also reported nausea, three episodes of vomiting, loss of appetite, dizziness, and unsteadiness resulting in a collapse at home. His partner additionally described transient confusion and slurred speech, raising concern for a posterior circulation stroke. 

On admission, he was febrile with a temperature of 40.1°C (104.2°F), blood pressure of 150/77 mmHg, heart rate of 70 beats/min, respiratory rate of 20 breaths/min, oxygen saturation of 94% on room air, and a National Early Warning Score (NEWS) of 3. Neurological examination demonstrated mild dysarthria and gait ataxia without focal motor weakness. Cranial nerve examination revealed subtle right facial asymmetry and slight tongue deviation; however, power was preserved (5/5) in all four limbs, with no sensory deficit or features of cauda equina syndrome. Initial laboratory investigations showed a C-reactive protein (CRP) level of 242 mg/L, a WBC count of 9.5 × 10⁹/L, and a lactate level of 1.7 mmol/L. The patient's laboratory investigations are summarized in Table 1.

**Table 1:** Initial bloods results CRP: C-reactive protein

Investigation	Result	NHS UK reference range
CRP	242 mg/L	<4 mg/L
WBC	9.5 × 10⁹/L	4.0-11.0 × 10⁹/L
Lactate	1.7 mmol/L	0.5-2.2 mmol/L

An urgent CT scan of the head demonstrated no acute intracranial haemorrhage or evidence of acute infarction. Chest radiography showed no focal consolidation or other acute cardiopulmonary abnormality. Given the combination of high-grade fever and elevated inflammatory markers, blood and urine cultures were obtained, and empirical intravenous broad-spectrum antibiotics and intravenous fluids were commenced for presumed sepsis of unknown origin. Stroke specialists considered the neurological symptoms more likely secondary to sepsis and advised reassessment if persistent neurological deficits remained after stabilization.

Despite antimicrobial therapy, the patient continued to experience intermittent pyrexia and persistent lower back pain [1]. A contrast-enhanced CT scan of the thorax, abdomen, and pelvis demonstrated no identifiable source of infection. Incidental findings included small subpleural pulmonary nodules within the lingula, with interval CT surveillance recommended after three months. Given the persistent back pain and absence of an alternative infective source, vertebral osteomyelitis [3] and spondylodiscitis remained important differential diagnoses.

Blood cultures obtained on admission subsequently grew MSSA in both bottles. Following microbiology consultation, antimicrobial therapy was changed to intravenous flucloxacillin, and repeat blood cultures were arranged to assess clearance of bacteremia. Owing to persistent MSSA bacteremia, transthoracic echocardiography (TTE) was performed, demonstrating no evidence of valvular vegetations, although infective endocarditis could not be definitively excluded.

**Figure 1:**
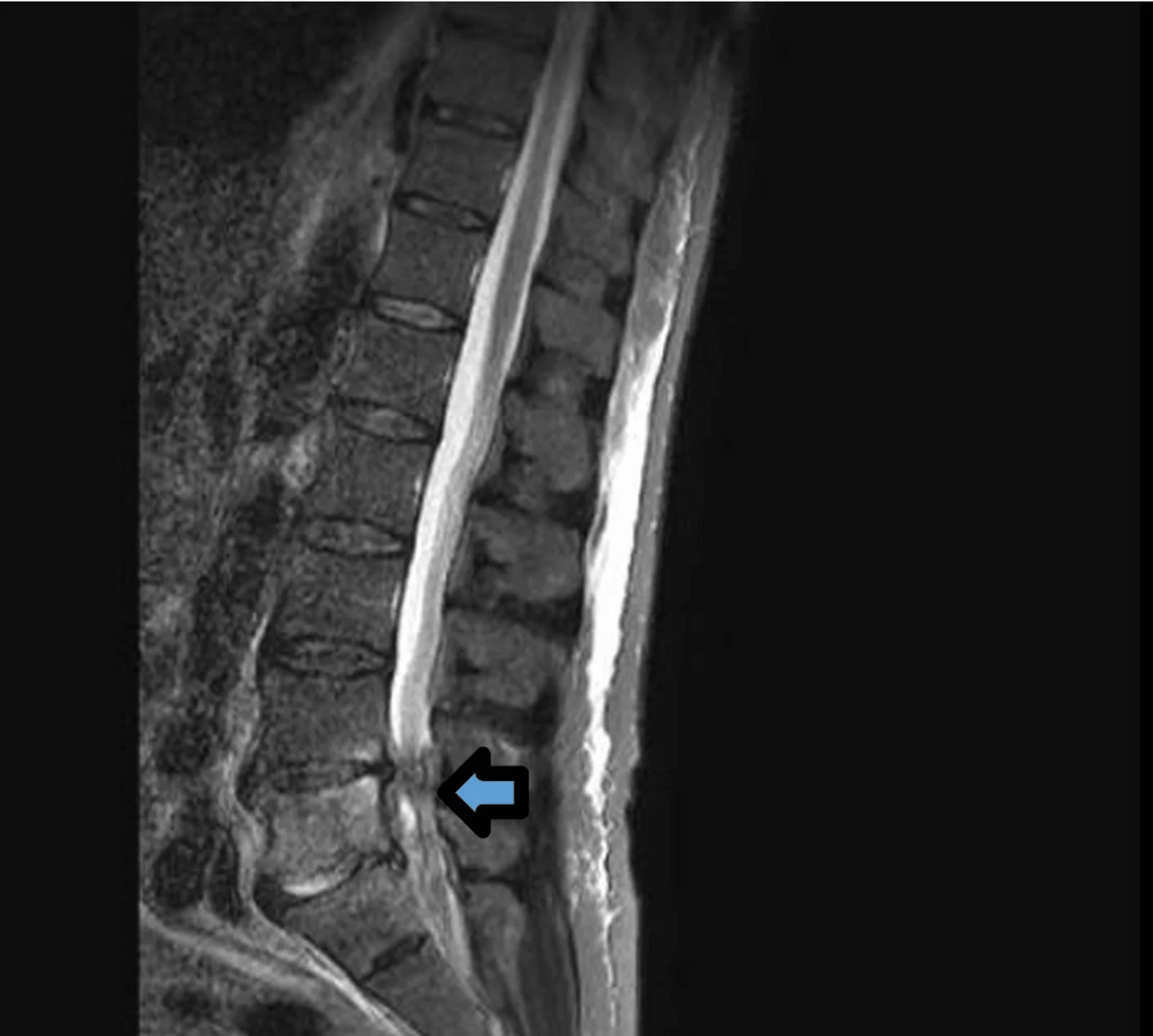
Initial whole-spine MRI showing multilevel degenerative cervical and lumbar spondylotic changes without evidence of discitis, vertebral osteomyelitis, or epidural abscess (blue arrow).

A whole-spine MRI performed early during admission (patient's first MRI of the whole spine) demonstrated multilevel degenerative cervical and lumbar spondylotic changes, including degenerative disc disease, but no convincing radiological evidence of discitis, vertebral osteomyelitis, or spinal epidural abscess (Figure 1). At that stage, there was no clear structural explanation identified for the patient's persistent lower back pain. However, given the presence of ongoing MSSA bacteremia, persistently elevated inflammatory markers, and continued clinical concern for a deep-seated spinal infective focus, vertebral infection could not be excluded despite the initial non-diagnostic imaging findings. The patient was therefore closely monitored, and further imaging was considered due to the persistent clinical suspicion.

**Figure 2:**
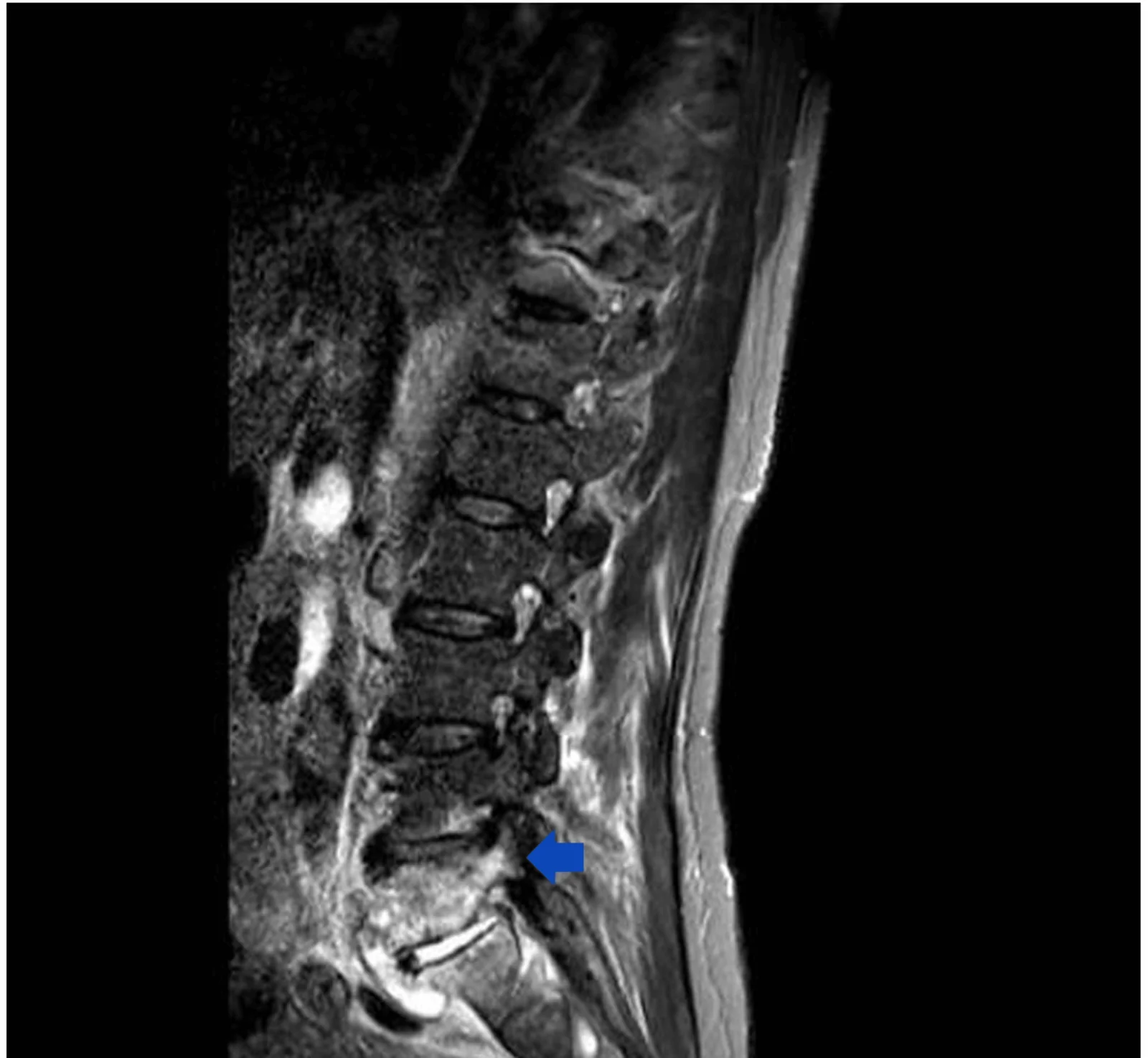
Repeat whole-spine MRI demonstrating fifth lumbar vertebra–first sacral vertebra (L5–S1) spondylodiscitis complicated by an anterior epidural abscess causing cauda equina compression, with associated anterior prevertebral fluid collection (blue arrow).

Approximately two weeks later, due to persistent MSSA bacteremia, ongoing lower back pain, and failure to identify an alternative source of infection, a repeat contrast-enhanced MRI of the lumbar spine was performed. This demonstrated new radiological evidence of fifth lumbar vertebra-first sacral vertebra (L5-S1) spondylodiscitis with an associated anterior epidural abscess causing significant compression of the cauda equina. Additionally, an anterior prevertebral fluid collection was identified, consistent with an extension of the infective process into the adjacent soft tissues. These findings confirmed the presence of a deep-seated spinal infection and provided an explanation for the persistent bacteremia (Figure 2).

Despite the significant radiological findings, the patient remained neurologically intact throughout his admission. He had preserved lower limb power, intact sensation, normal reflexes, and no bowel or bladder dysfunction. The case was discussed extensively in multidisciplinary meetings involving general medicine, microbiology, radiology, cardiology, and the regional spinal surgery team. Given the absence of objective neurological deficits, improving inflammatory markers, and gradual clinical improvement, the spinal multidisciplinary team recommended conservative management rather than surgical intervention.

During admission, the patient also developed a truncal vesicular rash. Dermatology review and viral PCR testing confirmed herpes simplex virus type 1 (HSV-1) infection, which was successfully treated with antiviral therapy. Repeat TTE demonstrated mildly impaired left ventricular systolic function (estimated left ventricular ejection fraction approximately 45%) without evidence of large valvular vegetations, and ramipril was initiated following cardiology review.

The patient's fever resolved, inflammatory markers progressively improved, and his back pain gradually became manageable with analgesia. Follow-up blood cultures demonstrated clearance of bacteremia. He remained neurologically stable throughout his hospital stay and regained independent mobility. Following multidisciplinary discussion, he was discharged under the Outpatient Parenteral Antimicrobial Therapy (OPAT) service to complete a six-week course of intravenous flucloxacillin, with arrangements for repeat contrast-enhanced lumbar MRI before completion of therapy.

## Discussion

Vertebral osteomyelitis and diagnostic challenges

Vertebral osteomyelitis, also termed spondylodiscitis when infection involves the intervertebral disc and adjacent vertebral endplates, is an uncommon but potentially severe infection of the spine. It represents approximately 3% to 5% of all cases of osteomyelitis and has become increasingly recognized due to improved imaging techniques and an aging population with increasing rates of diabetes, immunosuppression, and invasive procedures [1]. The infection usually occurs through hematogenous dissemination, with *S. aureus* being the predominant causative organism.

The clinical presentation of vertebral osteomyelitis is frequently non-specific, which contributes to delays in diagnosis. Persistent back pain is the most common symptom, while the classical triad of fever, back pain, and neurological deficit occurs in only a minority of patients. Delayed recognition may result in serious complications, including spinal deformity, neurological compromise, epidural abscess formation, and sepsis [1]. In our patient, the initial presentation was further complicated by transient neurological symptoms, including dysarthria and gait imbalance, which initially raised suspicion of a cerebrovascular event. However, negative neurological imaging combined with persistent systemic inflammation and ongoing bacteremia redirected the diagnostic approach towards an underlying infectious source.

Persistent MSSA bacteremia and metastatic infection

*Staphylococcus aureus *bacteremia is associated with significant morbidity and mortality due to its capacity for hematogenous dissemination and metastatic infection. Common metastatic complications include infective endocarditis, vertebral osteomyelitis, septic arthritis, deep abscess formation, and infection involving implanted prosthetic devices [1,2-8]. Persistent bacteremia despite appropriate antimicrobial therapy is an important predictor of complicated infection and should prompt clinicians to actively search for an uncontrolled source or metastatic focus. Studies have demonstrated that prolonged duration of bacteremia is associated with increased risk of metastatic complications and mortality [8,9].

In our patient, persistent MSSA bacteremia despite appropriate antimicrobial therapy raised suspicion of an occult deep-seated focus. This ultimately resulted in repeat spinal imaging and identification of L5-S1 spondylodiscitis complicated by epidural abscess formation. This case further supports published evidence that persistent SAB should prompt a thorough search for metastatic infection, particularly when no alternative source is identified, and blood cultures remain persistently positive despite appropriate antimicrobial therapy.

Limitations of early MRI and importance of repeat imaging

An MRI with gadolinium enhancement is considered the preferred imaging modality for diagnosing vertebral osteomyelitis and spinal epidural abscess because of its superior ability to demonstrate vertebral marrow involvement, disc-space infection, epidural extension, and paraspinal soft tissue abnormalities. The MRI provides excellent diagnostic accuracy, with reported sensitivity and specificity exceeding 90% in vertebral osteomyelitis [1-5]. However, early MRI may occasionally fail to demonstrate definitive infection, particularly during the initial inflammatory phase. Radiological abnormalities may evolve, and a negative early MRI should not exclude vertebral infection when clinical suspicion remains high. Current guidelines recommend repeating imaging when symptoms persist or when bacteremia remains unexplained [1]. In this case, the initial whole-spine MRI demonstrated only degenerative changes without evidence of infection. Persistent MSSA bacteremia and ongoing severe lower back pain prompted repeat contrast-enhanced MRI, which subsequently revealed L5-S1 spondylodiscitis, anterior epidural abscess, and prevertebral collection.

Our case closely mirrors the recommendations of the Infectious Diseases Society of America (IDSA) guideline [1], which advises repeat MRI within one to three weeks when the initial examination is negative but clinical suspicion remains high. While the MRI is highly sensitive for vertebral osteomyelitis, early imaging may fail to demonstrate characteristic inflammatory changes before infection becomes radiologically apparent. In our patient, repeat contrast-enhanced MRI performed approximately two weeks after the initial study confirmed L5-S1 spondylodiscitis with epidural abscess, highlighting the importance of serial imaging guided by clinical evolution rather than reliance on a single negative MRI examination.

Spinal epidural abscess and importance of early recognition

Spinal epidural abscess is an uncommon but potentially catastrophic complication of spinal infection. It occurs when infection spreads into the epidural space, causing compression of neural structures and potentially irreversible neurological injury. Risk factors include bacteremia, diabetes mellitus, immunosuppression, intravenous drug use, and spinal instrumentation [6].

The diagnosis of SEA requires a high index of clinical suspicion because neurological deficits may occur late and progression can be rapid. An MRI with contrast remains the investigation of choice because it accurately identifies epidural collections and defines the extent of disease [6-10]. Unlike many published cases in which spinal epidural abscess presents following neurological deterioration, our patient remained neurologically intact despite radiological evidence of cauda equina compression, allowing time for multidisciplinary assessment and careful treatment planning.

Conservative management versus surgical intervention

Management of vertebral osteomyelitis complicated by epidural abscess requires individualized decision-making involving infectious diseases specialists, microbiologists, radiologists, and spinal surgeons. Prolonged pathogen-directed antimicrobial therapy remains central to treatment; however, surgical intervention is required in selected patients [1,7,11]. Accepted indications for surgery include progressive neurological deficit, spinal cord or cauda equina compression with clinical deterioration, spinal instability, persistent bacteremia despite adequate antibiotic therapy, or failure of conservative treatment. Conversely, selected patients without neurological impairment may achieve favorable outcomes with antimicrobial therapy alone under close monitoring [7,11].

Despite radiological evidence of cauda equina compression in our patient, repeated neurological examinations remained normal, bacteremia cleared, inflammatory markers improved, and the spinal multidisciplinary team recommended conservative management with prolonged intravenous flucloxacillin through an OPAT pathway. This favorable outcome is consistent with published literature demonstrating that carefully selected patients without neurological deficits may be successfully managed with prolonged targeted antimicrobial therapy alone, provided that close neurological observation and multidisciplinary follow-up are maintained.

Clinical lessons and conclusion

This case highlights an important diagnostic challenge in the evaluation of suspected vertebral osteomyelitis and spondylodiscitis, i.e., a normal or non-diagnostic initial MRI does not completely exclude spinal infection when clinical suspicion remains high. Although MRI is the imaging modality of choice due to its high sensitivity for detecting vertebral infection and associated complications, radiological findings may evolve during the early stages of disease. Therefore, clinicians should avoid excluding vertebral osteomyelitis solely based on an initial negative MRI, particularly in patients with persistent SAB, ongoing focal back pain, or persistently elevated inflammatory markers.

In our patient, the initial whole-spine MRI performed early during admission showed only chronic degenerative changes without evidence of discitis or epidural abscess. However, the continued presence of MSSA bacteremia and persistent lower back pain maintained a high clinical suspicion for an occult spinal focus. Repeat contrast-enhanced MRI performed approximately two weeks later demonstrated L5-S1 spondylodiscitis complicated by an anterior epidural abscess and prevertebral collection, confirming the diagnosis. This progression illustrates that vertebral infection can be radiologically occult during the early phase and emphasizes the importance of serial imaging guided by clinical evolution rather than relying on a single negative investigation.

Additionally, this case reinforces the importance of considering persistent SAB as a potential marker of metastatic infection requiring systematic evaluation for deep-seated foci. Successful management requires close collaboration between multiple specialties, including microbiology, radiology, cardiology, general medicine, and spinal surgery. Despite the presence of radiological epidural involvement, conservative treatment was considered appropriate for our patient due to preserved neurological function, clinical improvement, microbiological clearance, and ongoing multidisciplinary review.

Overall, the key message from this case is that clinical suspicion should remain the driving factor in the diagnosis of vertebral osteomyelitis. A negative early MRI should not provide false reassurance when bacteremia and symptoms persist. A repeat contrast-enhanced imaging should be considered to identify evolving spinal infection and prevent potentially devastating neurological complications.

## Conclusions

Persistent SAB should always prompt consideration of an occult deep-seated infection, particularly in patients with persistent focal symptoms such as severe back pain. Although the MRI is the imaging modality of choice for vertebral osteomyelitis and spinal epidural abscess, early imaging may be falsely negative. In patients with ongoing bacteremia, rising inflammatory markers, or persistent symptoms, repeat MRI should be actively pursued when clinical suspicion remains high. 

The key clinical message from this case is that an initially negative MRI study should never provide false reassurance when there is persistent SAB and ongoing focal spinal symptoms. Clinical judgement should take precedence over a single negative investigation. When suspicion for vertebral osteomyelitis or spinal epidural abscess remains high despite an initially negative MRI, a repeat MRI should be performed within approximately two weeks, or earlier if there is clinical deterioration, as evolving infective changes may only become radiologically apparent over time.
